# Effects of different velocity loss thresholds on strength, neuromuscular adaptations, and muscle hypertrophy during bench press training in women

**DOI:** 10.5114/biolsport.2026.152347

**Published:** 2025-08-29

**Authors:** Luis Rodiles-Guerrero, Clara Cano-Castillo, Pedro Jesús Cornejo-Daza, Juan Sánchez-Valdepeñas, Borja Sañudo, Miguel Sánchez-Moreno, Beatriz Bachero-Mena, Fernando Pareja-Blanco

**Affiliations:** 1 Department of Human Movement and Sport Performance, University of Seville, Spain; 2 Science-Based Training Research Group, Physical Performance and Sports Research Center, Universidad Pablo de Olavide, Seville, Spain; 3 Faculty of Sport Sciences, Department of Sports and Computer Sciences, Universidad Pablo de Olavide, Seville, Spain; 4 Physical Education and Sports Department, Cardenal Spínola CEU Andalucía University, Bormujos, Sevilla, Spain; 5 Department of Physical Education and Sports, University of Seville, Seville, Spain

**Keywords:** Fatigue, Female athletes, Neural adaptations, Resistance training, Structural adaptations, Training volume

## Abstract

This study aimed to compare the effect of three velocity loss (VL) thresholds – 0% (VL0: one repetition per set), 25% (VL25), and 50% (VL50) – on strength gains, neuromuscular adaptations, and muscle hypertrophy during the bench press (BP) exercise. Forty-nine resistance-trained women were randomly assigned to three groups that followed an 8-week (16 sessions) BP training program using intensities ranging from 70% to 85% of 1-repetition maximum (1RM). Training groups differed in the VL achieved in each set (VL0, VL25, and VL50). Assessments performed before and after the training program included: 1) muscle thickness of the triceps brachii (TB); 2) maximal isometric test; 3) progressive loading test; and 4) fatigue test, all in the BP exercise. Electromyography signal was recorded from TB during these tests. Although all groups obtained significant gains in all strength variables, VL50 showed greater gains in 1RM strength and velocity against heavy loads than VL0 (group × time interaction: P < 0.001 and P = 0.04, respectively). Significant improvements in the force-time variables and electromyography amplitude (P = 0.01, with light loads) were observed exclusively in the VL25 group. Additionally, only VL50 exhibited significant increases in TB muscle thickness (P = 0.01). The greater the VL threshold, the more positive the effects on performance against heavy loads and muscle hypertrophy. Moderate VL thresholds improved the ability to produce force at high velocity and led to positive neuromuscular adaptations. Performing only one repetition per set was enough to improve strength in intermediate-trained women but was insufficient to maximize strength gains.

## INTRODUCTION

Resistance training is widely recognized as the most effective method for improving muscular strength across different populations [1–3]. Research on women also demonstrates the benefits of various resistance training methods [4–7]. Despite this, it is worth pointing out that most studies on resistance training have been carried out with male participants [8, 9], leading to training programs for women based on findings from male participants.

Recent technological advances have enabled resistance training to be monitored and have allowed for the real-time prescription of intensity and volume based on movement velocity. This approach, termed velocity-based training (VBT), is based on two key findings: i) the relationship between lifting velocity and relative intensity (expressed as a percentage of 1-repetition maximum, %1RM) [10]; and ii) the strong correlation between velocity loss (VL) and the percentage of repetitions completed (R^2^ = 0.88–1.00) in men [11–13] and women [14]. VBT allows practitioners to control intensity and volume effectively, both critical variables determining the level of effort exerted and, consequently, strength training outcomes [15, 16].

Previous research regarding the effects of VBT after a given training period in the bench press (BP) exercise has shown that applying VL thresholds of 25–50% can help maximize muscle hypertrophy, while lower VL thresholds (< 25%) evoked positive neuromuscular-related adaptations with lower mechanical and metabolic fatigue [17]. This outcome has been consistently observed regardless of training intensity, as similar results have been reported in male populations using heavy- (70–85% 1RM) [17], moderate- (55–70% 1RM) [18] and light- (40–55% 1RM) loads [19]. Indeed, these three studies have shown an inverted U-shaped relationship between the VL threshold and BP performance gains [17–19]. These findings suggest that 25–35% VL thresholds are optimal for maximizing 1RM gains in the BP exercise. Consequently, performing more fatiguing repetitions beyond this point does not lead to further gains and may even be detrimental to performance gains.

It is worth pointing out that all studies comparing the long-term training effects of different VL thresholds, except for one [20], have been conducted exclusively on resistance-trained men. As research has shown differences in neuromuscular characteristics and fatiguability between sexes [21, 22], the findings observed in men may not directly apply to women. Indeed, in the study by Rissanen et al. [20], men obtained similar strength improvements in the back squat and BP exercises following the 20% and 40% VL training programs. However, women demonstrated greater strength gains with the 40% VL protocol than the 20% VL. This suggests that it may be possible that women require a greater VL (i.e., higher within-set fatigue) than men to maximize strength and power development, especially in the BP exercise [20]. Therefore, in an attempt to gain further insight into the adaptations of resistance training provoked by different VL thresholds in upper body exercises in women, we aimed to compare the effects of 3 VBT programs in the BP exercise that differed in the VL allowed in each set (0% vs. 25% vs. 50%) on strength gains, neuromuscular adaptations, and muscle hypertrophy using intensities ranging from 70% to 85% 1RM. Based on previous literature [17–19], we hypothesized that VL thresholds between 25–50% will result in greater strength, muscle hypertrophy, and neuromuscular adaptations in women compared to lower thresholds.

## MATERIALS AND METHODS

### Experimental Design

An experimental research design was used to examine the effects of 3 different VL thresholds in the BP exercise in women: 0% (VL0: one repetition per set), 25% (VL25: 40–50% of the possible repetitions per set), and 50% VL (VL50: 75–85% of the possible repetitions per set) [14]. All groups performed an 8-week resistance training intervention (twice a week) using the BP exercise with the same relative intensity (70% to 85% 1RM). All participants were measured on two occasions: 72 h before (Pre-training) and 72 h after (Posttraining) the training intervention in two testing sessions (separated by 48 h). In the first testing session, the triceps brachii (TB) muscle thickness was measured. In the second testing session, a battery of tests in the BP exercise was performed as follows: 1) maximal isometric test, 2) progressive loading test, and 3) fatigue test. Sessions were performed in a research laboratory under the direct supervision of the investigators, at the same time of the day (± 1 h) and under the same environmental conditions (~20ºC and 60% humidity) for each participant. Each participant was verbally encouraged to exert maximum effort during all the testing and training sessions. One week before the Pretraining testing, all participants underwent two preliminary sessions during which they were familiarized with the testing equipment and exercise protocol.

### Subjects

Recruitment was performed through advertisements and community newsletters from September to October 2022. A total of 57 women volunteered to participate, all of whom had at least one year of experience in systematic resistance training involving the BP exercise. Upon enrollment, participants were asked about their menstrual cycle to ensure accurate data collection and documentation for potential future reference. They were also informed that if they experienced any discomfort during the Pre- or Post-training testing sessions that could affect performance, those sessions would be rescheduled. After recruitment, participants were allocated based on their 1RM into one of the three training groups: VL0, n = 19; VL25, n = 19; and VL50, n = 19. All participants signed informed consent forms before participating, and the local ethics committee approved the study design (Ref: 1547-N-19).

During the intervention, six women (one from VL0, one from VL25, and four from VL50) dropped out for reasons unrelated to the study. Moreover, two participants (one from VL0 and one from VL50) were excluded because they needed to complete 100% of the training sessions. The physical characteristics upon entering the study were: age = 21.4 ± 2.9 yr, height = 1.64 ± 0.06 m, and body mass = 61.3 ± 9.5 kg. The final number of participants in each group was VL0, n = 17; VL25, n = 18; and VL50, 14. The BP relative strength was 0.56, 0.53, and 0.52 kg · kg^−1^ for VL0, VL25, and VL50, respectively. These relative strength values correspond to an intermediate strength level in women, as Junior et al. [23] specified, who proposed performing the BP with at least 40–59.9% of body mass for this population.

### Testing procedures

#### Ultrasonography

**FIG. 1:**
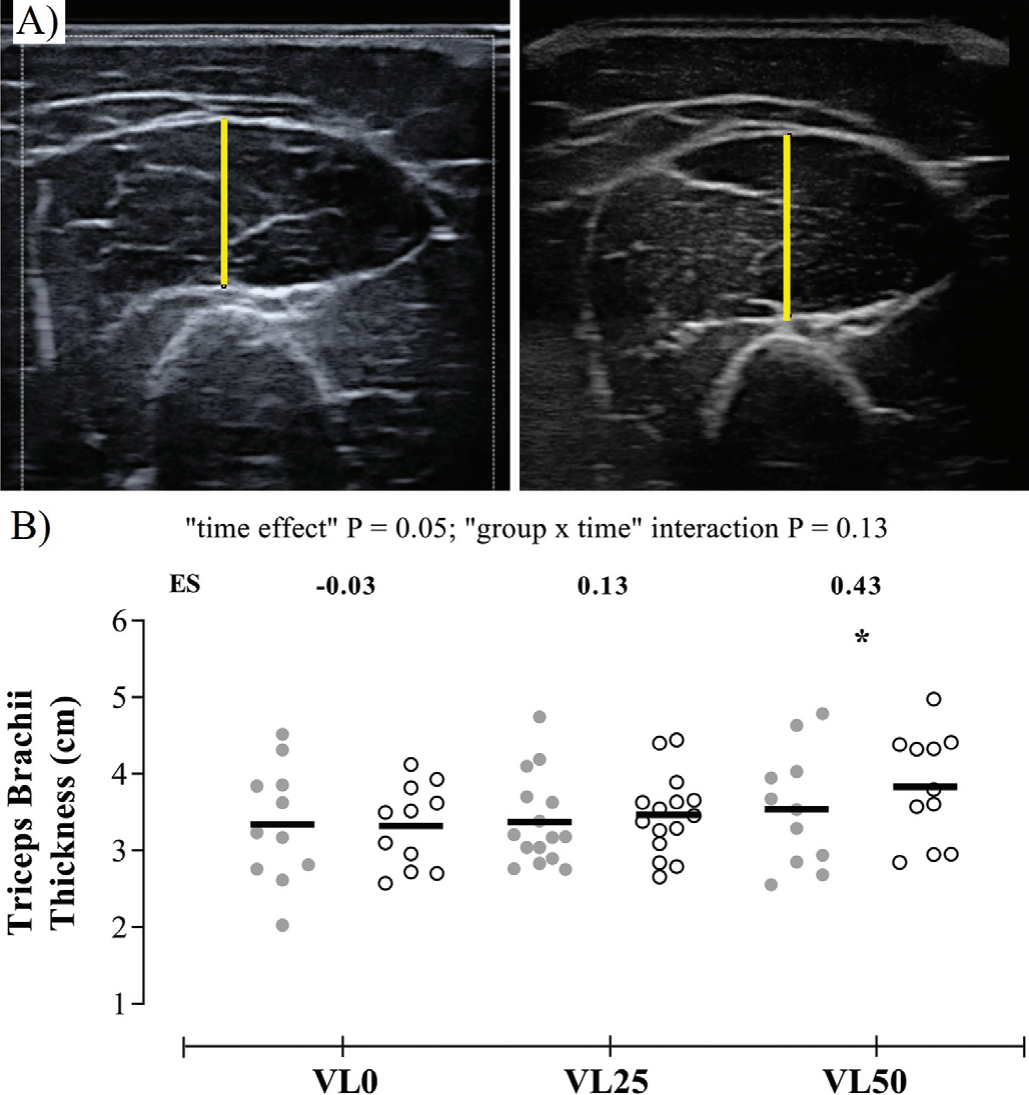
(A) Ultrasound image obtained from the triceps brachii muscle of a standard subject at Pre- and Post-training. The muscle thickness of the triceps brachii lateral head muscle is delimited by a yellow line. Due to excessive subcutaneous fat in the arm of some subjects, 12 subjects were removed from these analyses. (B) Changes produced in triceps brachii thickness from Pre- to Post-training for each group (N = 37). Grey and white circles indicate Pre- and Post-training values, respectively. VL0 indicates the group that trained with a mean velocity loss of 0% in each set (n = 11); VL25, the group that trained with a mean velocity loss of 25% in each set (n = 15); VL50, the group that trained with a mean velocity loss of 50% in each set (n = 11); ES, within-group effect size from Pre- to Post-training. Intragroup significant differences from Pre- to Post-training: *P < 0.05.

Muscle size was measured by muscle thickness of the TB Lateral head right arm using B-mode ultrasonography (Mylab 25, Esaote Biomedica, Italy) with a 50 mm, 5–12 MHz linear probe (Figure 1A). Before starting, participants remained in the supine position for 15 minutes. TB Lateral head measurement was taken at the midpoint between the medial epicondyle and the acromion with the participant lying face down, with the abduction of the shoulder and elbow joint ~90º. A custom-made device was placed under the elbow joint to avoid tissue deformation. Consistency in measurement sites across testing days was achieved by recording the probe positions on a transparent acetate sheet and using easily identifiable infiltrations of fatty and connective tissue as landmarks. All measurements were taken and analyzed using ImageJ 1.51j8 (NIH) by the same operator blinded to participant allocation. For each measurement, three images were captured, and the average of the first two was used for further analyses. If the coefficient of variation (CV) exceeded 5%, the third image was analyzed.

### Maximal isometric test

**FIG. 2:**
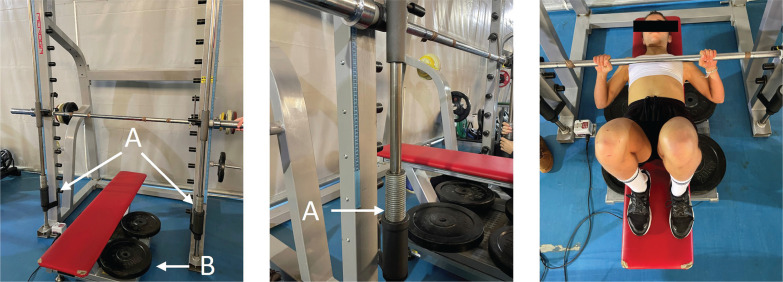
Setup used to assess the maximal isometric force. A) Telescopic bar holders to adjust the bar position of each participant. B) Dynamometric platform.

The test was performed on a Smith machine (Fitness Line, Peroga, Murcia, Spain) with the participants placed in the supine position on a bench (Bench Fitness Line, Peroga, Murcia, Spain) that was fitted onto a 0.8 × 0.8 m dynamometric platform (F-500, Ergotech, Murcia, Spain). Maximal isometric force (MIF) was measured with the bar placed 1 cm above the participant’s chest (elbow joint angle ~40º, considering full extension as 180º). Two telescopic bar holders with a precision scale were placed at the left and right sides of the Smith machine to precisely replicate the individual position of each participant between trials (Figure 2). Participants were instructed to produce as force as fast and hard as possible for about 5 s after the cue “ready, set, go!”. Two attempts with one minute rest were performed. Force data was collected at a sampling frequency of 1000 Hz and then filtered with specific software (T-Force System, Ergotech, Murcia, Spain). The following parameters were assessed during each trial: i) MIF; ii) maximal rate of force development (RFD_max_), which was calculated as the maximal slope of the forcetime curve in 20 ms time intervals; and iii) the average tangential slope of the force-time curve obtained over different time intervals (50, 100, 150, 200 and 400 ms from the onset of force production: RFD0–50, RFD0–100, RFD0–150, RFD0–200, and RFD0–400, respectively). The average value of each variable in the two attempts was used for further analyses.

### Progressive loading test and fatigue test

A Smith machine with no counterweight mechanism (Multipower Fitness Line, Peroga, Murcia, Spain) and a linear velocity transducer (T-Force System Ergotech, Murcia, Spain) were used for these tests. The velocity measures used in this study correspond to the mean velocity of the propulsive phase of each repetition (i.e., mean propulsive velocity, MPV), defined as that portion of the concentric action during which the measured acceleration is greater than the acceleration due to gravity (-9.81 m·s^−2^) [24]. Therefore, mean propulsive values avoid the breaking phase that negatively influences the computation of the mean concentric velocity with light and medium loads (i.e., until ~80% 1RM) (24). A ~1 s pause was imposed between the eccentric and concentric actions, with the bar resting on the chest, to minimize rebound effects and ensure consistent measurements [25]. A detailed description of this protocol has been provided elsewhere [17]. An average of 5.0 ± 1.2 increasing loads were used for each participant. Only the fastest repetition with each load was considered for subsequent analyses.

The warm-up consisted of two sets of 6 BP repetitions with 0.2 and 10 kg. To analyze the maximal unloaded velocity (V_0_), the first set consisted of 3 repetitions with a rigid plastic bar (bar weight < 0.2 kg). After this, the load was first set at 15 kg and progressively increased by 5 kg in subsequent sets until MPV was ≤ 0.40 m · s^−1^. At this point, the load was individually adjusted with smaller increments (from 2.5 to 1 kg) until the attained MPV was < 0.30 m · s^−1^ (~90% 1RM). The 1RM was determined based on each subject’s linear loadvelocity relationship (R^2^ = 0.99 ± 0.01). The 1RM was identified as the maximal load lifted at 0.17 m · s^−1^, which is the velocity corresponding to a 1RM in the Smith machine BP for strength-trained women [26]. Three repetitions were performed for light (> 0.80 m · s^−1^), two for medium (0.80–0.60 m · s^−1^), and only one for heavy (< 0.60 m · s^−1^) loads. Inter-set recoveries were 3, 4, and 5 minutes for light, medium, and heavy loads, respectively. The following variables derived from this test were used for analysis: a) 1RM load; b) V_0_, performed under free condition; c) average MPV attained against all absolute loads common to Pre- and Post-training (AV); d) average MPV attained against absolute loads that were lifted faster than 0.8 m·s^−1^ at Pre-training (AV > 0.8); and e) average MPV attained against absolute loads that were lifted slower than 0.8 m·s^−1^ at Pretraining (AV < 0.8). These variables were analyzed to examine the effects on the different parts of the load-velocity relationship. MPV attained with each %1RM was also analyzed. These data were obtained from the 1RM individual relative load-velocity relationship.

Five minutes after finishing the progressive loading test, the participants were required to complete as many repetitions as possible until reaching muscle failure with an absolute load corresponding to 70% 1RM at Pre-training. The execution technique and devices used were those described for the progressive loading test. The following variables were used for further analyses: a) maximal number of repetitions to failure (MNR), and b) average MPV attained against the same number of repetitions to Pre-training and Post-training (AV-MNR).

### Surface electromyography

Surface electromyography (EMG) was recorded from the TB muscle of the right arm during all tests, according to surface EMG recommendations for non-invasive muscle evaluation (27). An electrode was attached to the skin using double-sided adhesive tape and secured utilizing a mesh stretch covering. Electrode positions were recorded onto a transparent acetate (as described in the muscle thickness measurement) to ensure that the electrode was replaced precisely in the same location at Post-training assessments. EMG signals were recorded continuously using a parallel bar, bipolar surface electromyographic sensor wireless TrignoTM EMG system, with a bandwidth filter between 20 and 450 Hz ± 10% (Delsys Inc, MA, USA). Baseline noise was < 5 μV peak-to-peak, and sampling rate was 1,926 Hz. The raw data from the EMG were stored in digital format using EMG Works Acquisition software (Delsys Inc., MA, USA) and smoothed by root mean square (RMS) calculation using a moving window of 100 ms with an overlap of 99 ms. The RMS was calculated for: a) the two attempts during the isometric test, b) the fastest repetitions with each absolute load in the progressive loading test, and c) every repetition during the fatigue test. RMS values were normalized to those from maximal isometric contractions at Pre-training.

### Resistance training program

All participants completed two training sessions per week (48–72 h apart) for 8 weeks. All training groups used the same relative intensity, progressively increasing from 70% to 85% 1RM, number of sets (3), and inter-set recovery (4 minutes) throughout the 8-week intervention (Table 1). The difference between groups was the VL allowed in each training set. The VL threshold reached over each set was calculated as follows: 100 · (MPV_LAST_ – MPV_BEST_)/ MPV_BEST_. All repetitions were recorded using a linear velocity transducer (T-Force System, Ergotech, Murcia, Spain). The technical execution and settings were the same as those described in the progressive loading test, and the relative loads were determined from the individual load-velocity relationship obtained from this test (R^2^ = 0.99 ± 0.01). Therefore, absolute loads (i.e., kg) were adjusted for each training session to ensure the corresponding MPV matched (± 0.03 m · s^−^^1^) the prescribed %1RM. We used a range of 0.03 m · s^−^^1^ since it has been shown that this value is the smallest detectable change in MPV when using the T-Force System in the BP exercise on a Smith machine [28]. The warm-up preceding each training session was standardized for all training groups, as follows: a) 5 minutes of jogging at a self-selected easy pace, b) a set of 6 BP repetitions with 5–10 kg, followed by 3 sets of 6, 4 and 3 repetitions with loads of 40%, 50%, and 60% 1RM, respectively, for sessions 1–5, in which the training load was 70% 1RM. An additional set of two repetitions with 70% 1RM was added for sessions 6–14, in which the training load was 75%–80% 1RM. A final set of one repetition with 80% 1RM was added for sessions 15 and 16, in which the training load was 85% 1RM. A 3-minute rest between the warm-up sets was always used. The evolution of performance was measured using the absolute load (in kg) corresponding to 60% 1RM in every training session. This load was calculated based on the individual’s load-velocity relationship and chosen because it was used during the warm-up for all sessions.

**TABLE 1:** Descriptive characteristics of the 8-week velocity-based bench press training program performed by the three experimental groups

***Scheduled***	**Session 1**	**Session 2**	**Session 3**	**Session 4**	**Session 5**	**Session 6**	**Session 7**	**Session 8**

**Set × %1RM**	3 × 70	3 × 70	3 × 70	3 × 70	3 × 70	3 × 75	3 × 75	3 × 75

***Scheduled***	**Session 9**	**Session 10**	**Session 11**	**Session 12**	**Session 13**	**Session 14**	**Session 15**	**Session 16**

**Set × %1RM**	3 × 75	3 × 75	3 × 80	3 × 80	3 × 80	3 × 80	3 × 85	3 × 85


***Actually Performed***	**Fastest MPV** (m · s^−1^)		**Slowest MPV** (m · s^−1^)	**MPV all reps** (m · s^−1^)		**Mean VL** (%)	**Total Rep**	

VL0	0.50 ± 0.03		0.46 ± 0.03^25,50^	0.48 ± 0.03^25,50^		0^25,50^	48^25,50^	

VL25	0.51 ± 0.04		0.33 ± 0.03^50^	0.42 ± 0.04^50^		26.0 ± 1.8^50^	182.11 ± 31.42^50^	

VL50	0.51 ± 0.04		0.21 ± 0.02	0.37 ± 0.03		50.8 ± 1.9	308.5 ± 59.48	

***Actually Performed***	**Average rep per set in all sessions**		**Rep per set with 70% 1RM**	**Rep per set with 75% 1RM**		**Rep per set with 80% 1RM**	**Rep per set with 85% 1RM**	

VL0	1^25,50^		1^25,50^	1^25,50^		1^25,50^	1^25,50^	

VL25	3.8 ± 0.6^50^		4.9 ± 0.8^50^	3.6 ± 0.8^50^		3.2 ± 0.7^50^	2.5 ± 0.5^50^	

VL50	6.4 ± 1.2		8.1 ± 1.3	6.7 ± 1.7		5.2 ± 1.2	4.0 ± 0.9	

Data are mean ± standard deviation. VL0: Group that trained with a mean velocity loss of 0% in each set (n = 17); VL25: Group that trained with a mean velocity loss of 25% in each set (n = 18); VL50: Group that trained with a mean velocity loss of 50% in each set (n = 14). VL: Magnitude of velocity loss expressed as percent loss in mean repetition velocity from the fastest (usually first) to the slowest (last one) repetition of each set; MPV: Mean Propulsive Velocity; Fastest MPV: Average of the fastest repetitions measured in each session (this value represents the average intensity, %1RM, achieved during the training program); Slowest MPV: Average of the slowest repetitions measured in each session; MPV all reps: Average MPV attained during the entire training program; Mean Velocity Loss: Average velocity loss attained during the entire training program; Total rep: Total number of repetitions performed during the training program; Average rep per set in all sessions: average number of repetitions performed in each set; Rep per set with a given %1RM: average number of repetitions performed in each set with each of the loads used (70, 75, 80 or 85% 1RM). Statistically significant differences (P < 0.001) with: VL25: ^25^; VL50: ^50^.

### Statistical analysis

Values are reported as mean ± standard deviation (SD). Withinsession absolute reliability was measured by the CV. Within-session relative reliability was calculated using the intraclass correlation coefficient (ICC) with a 95% confidence interval (CI), using the 1-way random effects model. Normality and homoscedasticity were verified with Shapiro-Wilk and Levene´s tests, respectively. Data were analyzed using a 3 × 2 factorial ANOVA, one inter-group (VL0 vs. VL25 vs. VL50) and one within-group (Pre- vs. Post-training) factor. When significant interactions or effects were found, Bonferroni´s post-hoc comparisons were conducted. Statistical significance was established at the P ≤ 0.05 level. In addition, within-group effect size (ES) values were calculated using Hedge’s g on the pooled SD (29) using a purpose-built spreadsheet. The rest of the statistical analyses were performed using SPSS software version 23.0 (SPSS Inc., Chicago, IL, USA). Figures were designed using SigmaPlot 12.0 (Systat Software Inc., San Jose, CA, USA).

## RESULTS

### Training program

**FIG. 3:**
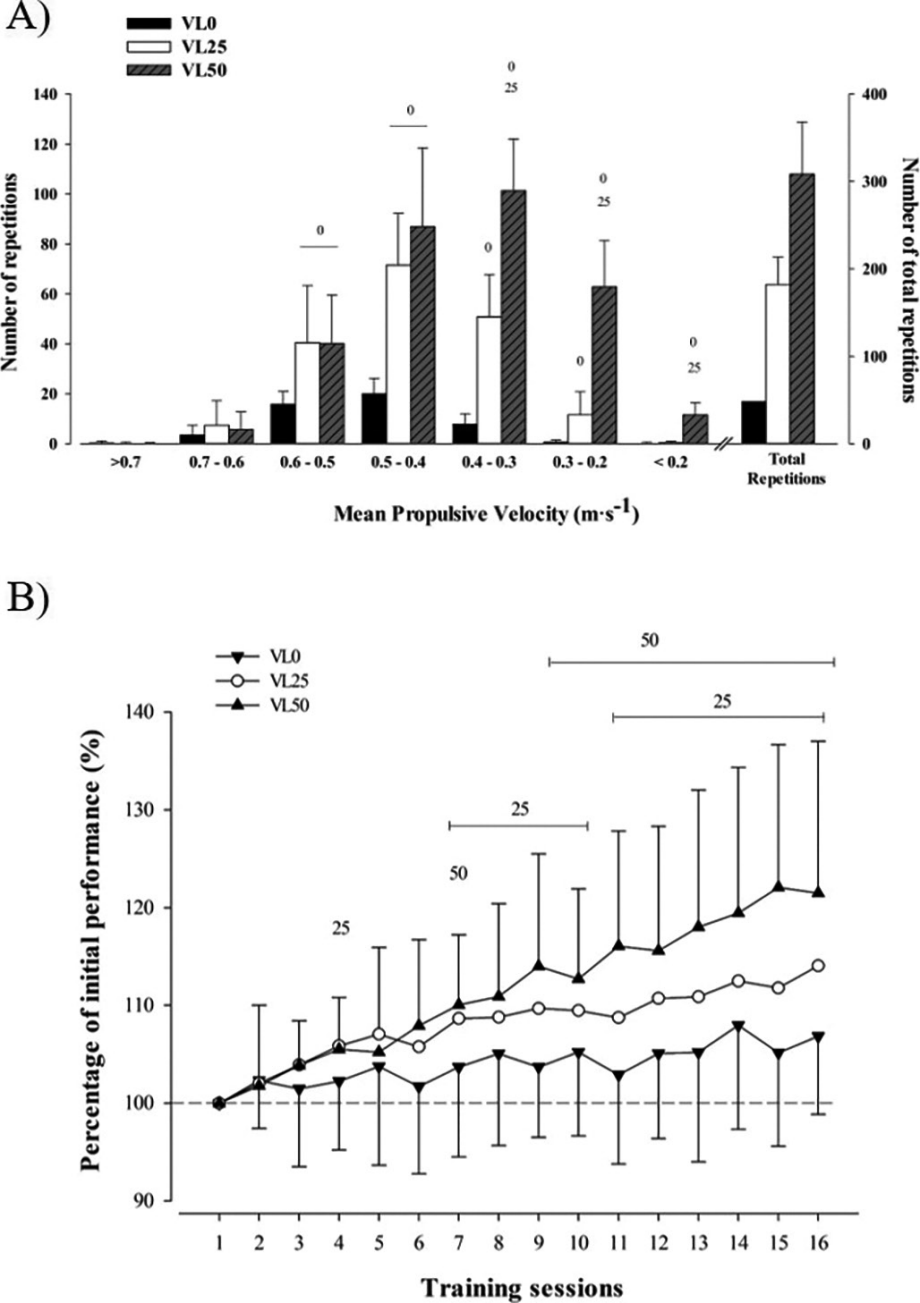
(A) Number of repetitions performed in different velocity ranges and total number of repetitions completed during the training program by three training groups. Between groups significant differences (P < 0.05) with respect to: VL0: 0; VL25: 25; and VL50: 50. (B) Evolution of performance in each training session was expressed as the percentage of the first training session level for three experimental groups. The numbers 0, 25, or 50 indicate the session from which the respective group significantly improved (P < 0.05) compared to their first session values. Data are mean ± standard deviation, N = 49. VL0: group that trained with a mean velocity loss of 0% in each set (n = 17); VL25: group that trained with a mean velocity loss of 25% in each set (n = 18); VL50: group that trained with a mean velocity loss of 50% in each set (n = 14).

Descriptive characteristics of the training performed by the three groups are reported in Table 1. The average of the fastest repetition measured in each session, representing the intensity (%1RM) lifted in each training session, did not differ between groups. The mean velocity (MPV all reps) attained during the training program decreased as the VL increased (P < 0.001). Moreover, the training volume (i.e., the total number of repetitions performed during the training program, average of repetitions per set in all sessions, and average of repetitions per set performed with each %1RM) increased as the magnitude of VL increased (P < 0.001). Participants reached muscle failure during 1 (85% 1RM) and 4 sets (80–85% 1RM) for VL25 and VL50, respectively. The repetitions performed in the different velocity ranges are shown in Figure 3A. Figure 3B shows the progression of performance in every training session.

### Test reliability values

Normality and homoscedasticity criteria were met for all variables. The reliability values of the different variables analyzed are shown in Table 2. No significant differences between groups were observed for any variable at Pre-training (P > 0.05).

**TABLE 2:** Relative (ICC with 95% CI) and absolute (CV) reproducibility of different variables analyzed

Parameter	ICC (95% CI)	CV, %
MIF, N	0.97 (0.94–0.98)	5.3
RFD_max_, N·s^−1^	0.93 (0.87–0.96)	15.7
RFD_0–50_, N·s^−1^	0.92 (0.83–0.96)	21.3
RFD_0–100_, N·s^−1^	0.90 (0.81–0.95)	17.4
RFD_0–150_, N·s^−1^	0.90 (0.79–0.95)	15.2
RFD_0–200_, N·s^−1^	0.89 (0.78–0.94)	14.7
RFD_0–400_, N·s^−1^	0.95 (0.90–0.97)	9.3
TB RMS, mV	0.98 (0.96–0.99)	9.0
TB LaH, cm	1.00 (0.99–1.00)	1.8
V_0_, m·s^−1^	0.92 (0.87–0.95)	5.6
MPV, m·s^−1^	0.98 (0.97–0.99)	4.5

Abbreviations: CV, coefficient of variation; ICC, intraclass correlation coefficient; CI, confidence interval; MIF, maximal isometric force; RFD, rate of force development; RFDmax, maximal RFD; RFD_0–50_, RFD from the onset of force production to 50 ms; RFD_0–100_, RFD from the onset of force production to 100 ms; RFD_0–150_, RFD from the onset of force production to 150 ms; RFD_0–200_, RFD from the onset of force production to 200 ms; RFD_0–400_, RFD from the onset of force production to 400 ms; TB RMS, maximal root mean squared value registered during the maximal voluntary isometric contraction in the triceps brachii (TB); TB LaH, TB lateral head muscle thickness; V_0_, maximal unloaded velocity; MPV, mean propulsive velocity with a common absolute load (15 kg). Note: N = 49.

### Triceps brachii hypertrophy

No significant group × time interaction was noted for the muscle thickness, while a significant overall time effect was reported (P = 0.048). Only VL50 showed significant increases in the thickness of TB (P = 0.01, Figure 1B).

### Maximal isometric test

No significant group × time interactions were noticed in the mechanical variables (all P > 0.05) (Table 3). Significant time effects were observed for MIF (P < 0.001) and RFD_max_ (P = 0.01), with all groups showing increased MIF (P < 0.001–0.05). However, significant gains were found in RFD_max_ (P = 0.04), RFD_0–100_ (P = 0.05), and RFD_0–200_ (P = 0.03) exclusively in the VL25 group.

**TABLE 3:** Changes in selected performance variables from Pre- to Post-training for each group.

	VL0			VL25			VL50			P-value	P-value

	Pre	Post	ES	Pre	Post	ES	Pre	Post	ES	time effect	group × time
**MIF**(N)	424.3 ± 97.0	460.1 ± 100.7^*^	0.41	387.7 ± 68.4	445.2 ± 63.0^**^	0.66	419.6 ± 94.6	467.0 ± 85.0^**^	0.54	< 0.001	0.63

**RFD_max_**(N · s^−1^)	2512.3 ± 941.2	3039.9 ± 1304.6	0.43	2372.8 ± 1013.6	3023.4 ± 1625.5^*^	0.53	2622.4 ± 1261.4	2819.3 ± 986.6	0.16	0.01	0.58

**RFD_0–50_**(N · s^−1^)	1613.7 ± 874.4	1810.1 ± 1122.1	0.19	1632.7 ± 750.2	2039.2 ± 1317.8	0.39	1695.9 ± 1057.0	1680.5 ± 952.2	-0.01	0.18	0.50

**RFD_0–100_**(N · s^−1^)	1704.1 ± 602.0	1784.1 ± 815.2	0.10	1573.6 ± 614.8	1945.1 ± 863.8^*^	0.49	1706.8 ± 901.1	1711.1 ± 692.0	0.01	0.17	0.35

**RFD_0–150_**(N · s^−1^)	1560.9 ± 505.9	1648.3 ± 645.0	0.16	1447.1 ± 436.3	1641.1 ± 543.6	0.35	1574.7 ± 615.3	1586.2 ± 529.8	0.02	0.17	0.57

**RFD_0–200_**(N · s^−1^)	1315.3 ± 414.0	1413.1 ± 537.9	0.22	1181.1 ± 307.0	1385.4 ± 402.7^*^	0.46	1363.4 ± 490.4	1370.5 ± 439.3	0.02	0.07	0.36

**RFD_0–400_** (N · s^−1^)	827.4 ± 243.2	868.2 ± 274.0	0.17	744.4 ± 170.6	832.8 ± 211.6	0.37	827.6 ± 238.3	833.3 ± 271.8	0.02	0.14	0.53

Data are mean ± standard deviation. N = 46. VL0: group that trained with a mean velocity loss of 0% in each set (n = 17); VL25: group that trained with a mean velocity loss of 25% in each set (n = 18); VL50: group that trained with a mean velocity loss of 50% in each set (n = 14). MIF: maximal isometric force; RFDmax: maximal rate of force development (RFD); RFD_0–50:_ RFD from the onset of force production to 50 ms; RFD_0–100:_ RFD from the onset of force production to 100 ms; RFD_0–150:_ RFD from the onset of force production to 150 ms; RFD_0–200:_ RFD from the onset of force production to 200 ms; RFD_0–400:_ RFD from the onset of force production to 400 ms. ES: effect size from Pre to Post-training. Intra-group significant differences from Pre to Post-training:

* P ≤ 0.05,

** P≤ 0.01.

### Progressive loading and fatigue test

Resistance training-induced adaptations in dynamic BP performance are reported in Table 4. A significant group × time interaction was found for 1RM (P < 0.001) and AV < 0.8 (P = 0.04). Moreover, significant time effects were observed for all performance parameters analyzed (all P < 0.001) except for V_0_. All groups showed significant gains in 1RM, AV, AV < 0.8, AV > 0.8, MNR, and AV-MNR (P < 0.05–0.001). Significantly greater AV and AV < 0.8 values were observed for the VL50 group compared to VL0 at Post-training (P < 0.05).

**TABLE 4:** Changes in selected performance variables from Pre- to Post-training for each group.

	VL0			VL25			VL50			P-value	

	Pre	Post	ES	Pre	Post	ES	Pre	Post	ES	time effect	group × time
**1RM** (kg)	32.8 ± 7.4	35.8 ± 7.7^***^	0.38	32.5 ± 7.9	36.1 ± 7.8^***^	0.45	33.5 ± 8.7	40.6 ± 7.7^***^	0.88	< 0.001	< 0.001

**V_0_** (m · s^−1^)	1.80 ± 0.14	1.84 ± 0.16	0.18	1.76 ± 0.24	1.75 ± 0.29	-0.06	1.80 ± 0.12	1.75 ± 0.21	-0.25	0.73	0.37

**AV** (m · s^−1^)	0.53 ± 0.08	0.63 ± 0.09^***^	0.85	0.56 ± 0.14	0.66 ± 0.09^***^	0.91	0.59 ± 0.12	0.75 ± 0.07^***^0	1.44	< 0.001	0.13

**AV < 0.8** (m · s^−1^)	0.45 ± 0.03	0.55 ± 0.09^***^	1.35	0.45 ± 0.06	0.57 ± 0.07^***^	1.61	0.46 ± 0.06	0.65 ± 0.08^***^0	2.40	< 0.001	0.04

**AV** ≥ **0.8** (m · s^−1^)	0.90 ± 0.08	0.98 ± 0.10^**^	0.95	0.93 ± 0.08	0.99 ± 0.07^**^	0.78	0.96 ± 0.07	1.01 ± 0.07^*^	0.63	< 0.001	0.81

**MNR** (rep)	11.8 ± 3.8	16.7 ± 4.2^***^	1.25	13.2 ± 2.9	18.4 ± 3.9^***^	1.36	12.7 ± 2.4	20.9 ± 4.4^***^	2.08	< 0.001	0.08

**AV-MNR** (m · s^−1^)	0.36 ± 0.05	0.50 ± 0.09^***^	1.64	0.37 ± 0.05	0.49 ± 0.12^***^	1.52	0.37 ± 0.04	0.56 ± 0.09^***^	2.25	< 0.001	0.24

Data are mean ± SD, N = 46. VL0: group that trained with a mean velocity loss of 0% in each set (n = 17); VL25: group that trained with a mean velocity loss of 25% in each set (n = 18); VL50: group that trained with a mean velocity loss of 50% in each set (n = 14). 1RM: one-repetition maximal in bench press exercise. V_0_: maximal unloaded velocity; AV: average MPV attained against all absolute loads common to Pre- and Post-training; AV ≥ 0.8: average MPV attained against absolute loads that were moved faster than 0.8 m · s^−1^ at Pre-training; AV < 0.8: average MPV attained against absolute loads that were moved slower than 0.8 m · s^−1^ at Pre-training; MNR: maximal number of repetitions in the fatigue test; AV-MNR: average MPV attained against the same number of repetitions to Pre-training and Post-training in the fatigue test. Intra-group significant differences from Pre to Post-training:

* P ≤ 0.05,

** P ≤ 0.01,

**** P ≤ 0.001.

### Changes in the relative load-velocity relationship

**FIG. 4:**
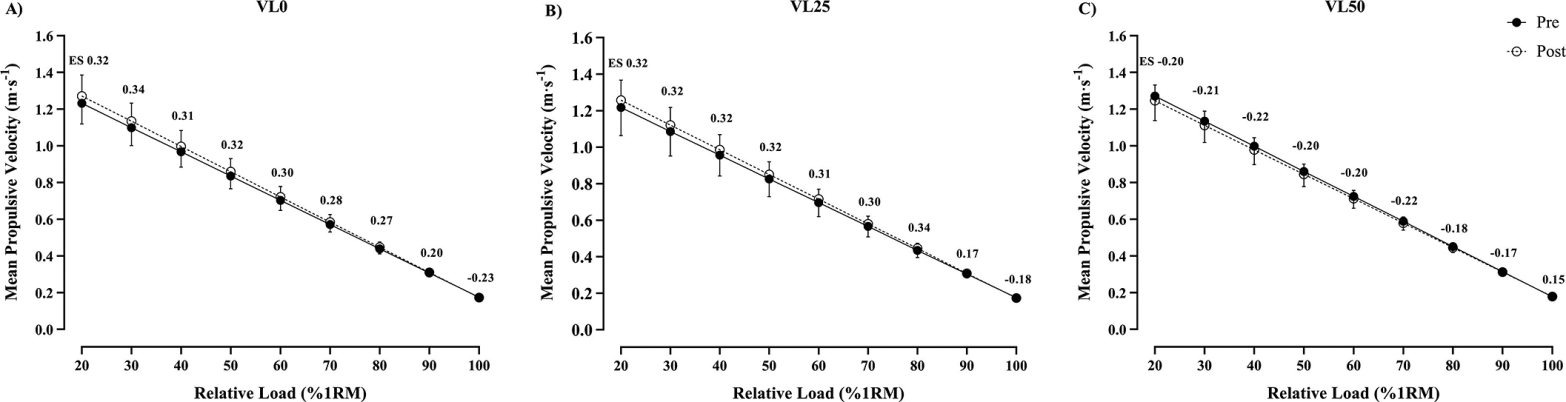
Changes in selected individual load-velocity relationship from Pre- to Post-training for the bench press training programs with different velocity loss thresholds. Data are expressed as means ± standard deviation, N = 49. VL0: group that trained with a mean velocity loss of 0% in each set (n = 17); VL25: group that trained with a mean velocity loss of 25% in each set (n = 18); VL50: group that trained with a mean velocity loss of 50% in each set (n = 14). ES, within-group effect size from Pre- to Post-training.

Figure 4 shows the Pre-Post change in the load-velocity relationship. No significant interactions were observed. VL0 and VL25 groups obtained improvements in the MPV attained from 20% to 90% 1RM (Higer ES values). However, VL50 obtained a decreased MPV with loads between 20 to 90% 1RM.

### EMG adaptations

**FIG. 5:**
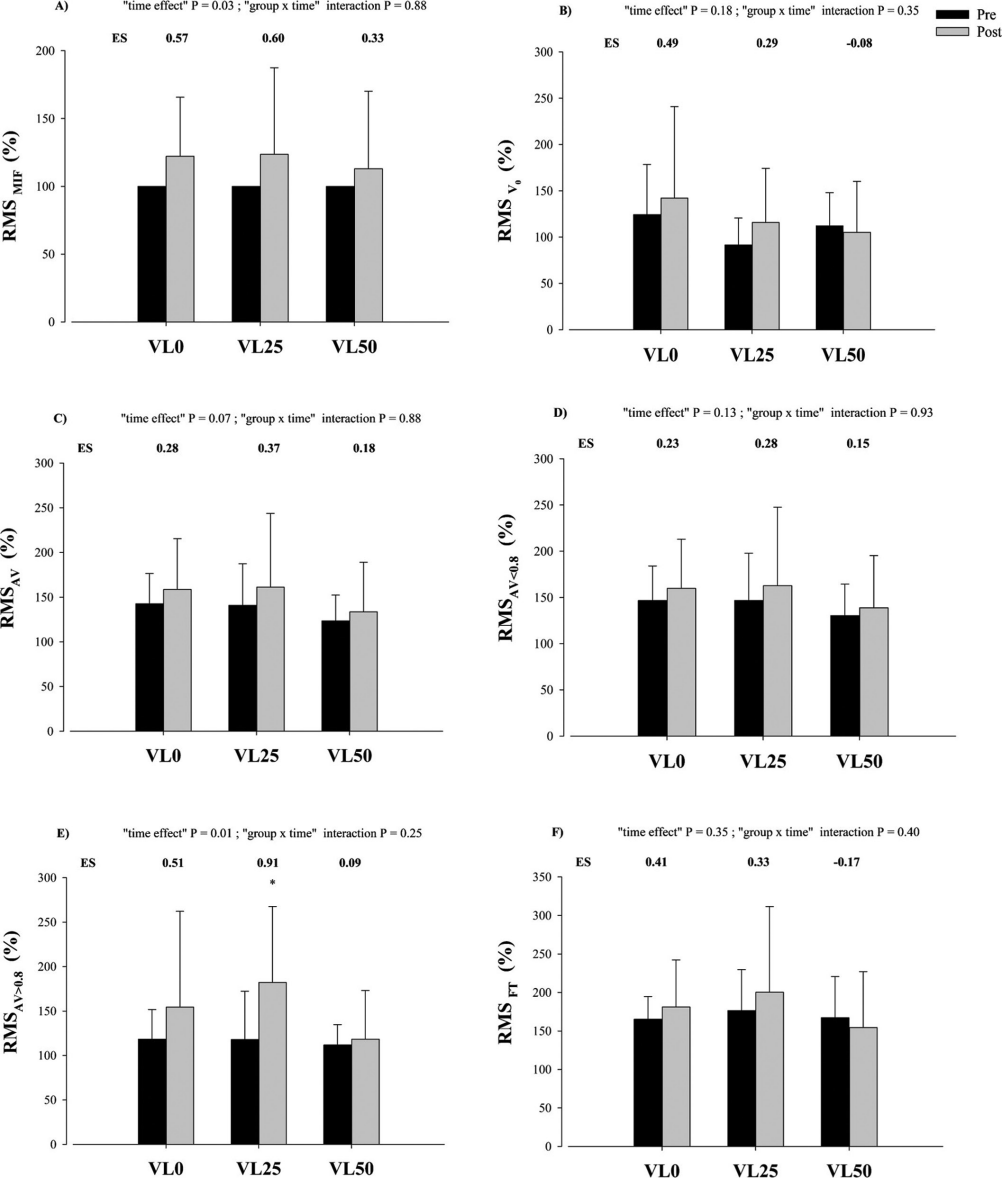
Changes produced in normalized root mean square (RMS) value registered from Pre- to Post-training for each group in: maximal voluntary isometric contraction force (RMS_MIF_, A); maximal unloaded velocity (RMSv_0,_ B); average mean propulsive velocity (MPV) attained against absolute loads common to Pre- and Post-training (RMS_AV_; C); average MPV attained against absolute loads that were moved slower than 0.8 m · s^−1^ at Pre-training (RMS_AV < 0.8_; D); average MPV attained against absolute loads common to Pre and Post that were moved faster than m · s^−1^ at Pre-training (RMS_AV > 0.8_; E); and average MPV attained against the same number of repetitions to Pre-training and Post-training in the fatigue test (RMS_FT_; F); following the resistance training programs against 70%– 85% 1RM in the bench press exercise. Data are expressed as means ± standard deviation, N = 49. VL0: group that trained with a mean velocity loss of 0% in each set (n = 17); VL25: group that trained with a mean velocity loss of 25% in each set (n = 18); VL50: group that trained with a mean velocity loss of 50% in each set (n = 14). ES, within-group effect size from Pre- to Posttraining.

Descriptive data and ES values for each neuromuscular parameter analyzed are reported in Figure 5. No significant group × time interactions were noted in the EMG variables. A significant overall time effect was observed for RMS MIF (P = 0.03) and RMS_AV > 0.8_ (P = 0.01). The VL25 intervention significantly increased RMS_AV > 0.8_ (P = 0.01). No further significant changes were detected in any EMG variable.

## DISCUSSION

The present study was designed to compare the effects on strength gains, neuromuscular adaptations, and muscle hypertrophy of 3 VBT programs in the BP exercise that differed in the VL allowed in each set (0% vs. 25% vs. 50%) in intermediate resistance-trained women. Our results point out that women’s response to resistance training may vary from men’s (17–19), particularly in terms of the volume and fatigue required for strength gains. The main findings of this investigation were: i) the greater the VL threshold the more positive the effects on performance against heavy loads and muscle hypertrophy; ii) moderate VL thresholds (i.e., VL25) improved the ability to produce force at short time intervals (i.e., RFD_max_, RFD_100_, and RFD_200_) and led to positive neuromuscular adaptations during highvelocity lifting (i.e. increased RMS_AV > 0.8_); and iii) performing only one repetition per set (i.e., VL0) using 70–85% 1RM, twice weekly, was enough to improve strength in intermediate-trained women but was insufficient to maximize strength gains, particularly for heavy loads.

After completing an 8-week BP-VBT program involving 16 training sessions with loads ranging from 70% to 85% 1RM, all groups experienced significant improvements in strength performance. Likewise, higher VL thresholds (i.e., VL50) maximized strength gains with heavy loads. These findings partially align with a previous VBT study using the BP exercise with the same relative loads (70%–85% 1RM) carried out in resistance-trained young men, where VL25 was the most effective (17). However, our results support recent research in women [20], suggesting that higher VL thresholds (i.e., VL40) improve strength gains [20]. Interestingly, Rissanen et al. [20] did not find additional benefits of training with higher VL thresholds in men. These authors conclude that women may require more within-set fatigue to maximize strength adaptations, highlighting the need for higher training volumes than men [20]. Several key considerations emerge when examining sex-specific responses to VBT, particularly concerning the relationship between VL thresholds and the %Rep relative to the MNR. Firstly, the association between VL and %Rep appears to differ between sexes. In a study by Bachero-Mena et al. [14], it was observed that, for a given VL threshold, women completed a lower %Rep compared to those reported for men [30]. This suggests that the same VL percentage corresponds to a lower relative effort in women, potentially necessitating higher VL thresholds to elicit comparable training stimuli and adaptations. Secondly, the VL thresholds compared in the study by Rissanen et al. [20] (20% vs. 40%) differ from those in the current study (25% vs. 50%). Bachero-Mena et al. [14] demonstrated that VL thresholds of 20%, 25%, 40%, and 50% correspond to approximately 30–40%, 40–50%, 60–70%, and 75–85% of MNR, respectively, in the BP exercise. Previous research has indicated that VL thresholds of 25–35% are optimal for maximizing 1RM gains in the BP exercise in men [17–19]. However, these VL thresholds correspond to 50–60% of MNR in men [30], which aligns with a 40% VL threshold in women [14]. These findings underscore the importance of considering sex-specific responses in VBT. Implementing individualized VL thresholds that account for these differences may optimize training outcomes for women.

Remarkably, the benefits produced by higher VL thresholds disappear when lifting loads at high velocities (i.e., AV > 0.8 and V_0_). In these cases, the lower fatigue protocols produce more favorable outcomes, as ES values indicate, suggesting that lower VL thresholds may improve performance in high-velocity actions. Martínez-Cantón et al. [31] reported a relationship (r = 0.59) between the total repetitions performed during the resistance training program and muscle CaMKII response. These authors also observed a link between the rise in CaMKII, reduced IIX muscle fiber type, and increased muscle mass [31]. Likewise, a previous study on the squat exercise demonstrated that a VL40 threshold led to a decrease in the percentage of IIX muscle fibers, while this percentage remained unchanged in the VL20 group [32]. A faster muscle fiber phenotype offers an advantage in high-velocity movements, particularly under light loads or unloaded conditions [33, 34]. The fact that the low and moderate VL threshold protocols may have preserved their IIX muscle fibers could explain the potential better adaptations observed in these protocols during high-velocity actions. In this regard, a previous study found a significant correlation (r^2^ = 0.49) between the vastus lateralis type II fiber area and the RFD_0–50_ of the knee extensor muscles [35], suggesting that individuals with larger type II muscle fibers tend to have better early-phase force production. Intriguingly, although all groups improved their MIF, only the VL25 intervention enhanced the ability to produce force quickly (i.e., RFD_max_, RFD_0–100_, and RFD_0–200_). It has been suggested that the improvement in the contractile RFD may be influenced by the level of neural activation [36]. Indeed, the VL25 intervention was the only group to demonstrate positive neuromuscular adaptations during high-velocity lifting, such as increased RMS_AV > 0.8_. Previous BP-VBT studies have shown that the degree of VL induced during the set is a critical factor in modulating neuromuscular adaptations that occur during resistance training in trained men [17, 18, 37]. These studies displayed that moderate VL thresholds (i.e., 15–25%) provide an optimal training stimulus to maximize neuromuscular adaptations, while higher VL thresholds (i.e., ≥ 50%) could induce negative neuromuscular adaptations. Hence, the results obtained in the present study confirm the assumption that a moderate VL threshold (i.e., VL25) within the set is an optimal stimulus for adaptations in muscle excitation and to improve the RFD for intermediate resistance-trained women, as well as occurs in men [18].

Strength deficit refers to the relative difference in force production between the force exerted against the 1RM load and the force applied at any submaximal relative load [38]. This concept is important as it offers insights into how different types of resistance training affect the capacity to apply force across various relative loads. In practice, an improvement (i.e., decrease) in strength deficit corresponds to enhanced velocity at a given relative load, indicating an increased capacity to apply force at that load. In the present study, VL0 and VL25 increased MPV from 20% to 90% 1RM, while VL50 decreased MPV with loads between 20% and 90% 1RM. In agreement with other strength-derived variables, lower fatigue protocols yield more favorable outcomes than more fatiguing resistance training interventions. The unintentional slow repetitions conducted by VL50 may be behind these findings. As previously mentioned, the total repetitions performed during a resistance training program, CaMKII phosphorylation, and the reduction of IIX muscle fiber types appear to be interconnected [31], which may impair force production at high velocities [33, 34].

Conversely, the higher number of repetitions completed by VL50, along with the higher metabolic and hormonal responses typically observed in these protocols [15], may explain the greater muscle hypertrophy induced by these protocols. In this regard, it has been shown that muscle size favors force production against heavy loads, that is, at low velocities [39]. Therefore, the higher strength gains observed for VL50 during heavy lifts may be explained, at least partially, by the muscle hypertrophy observed in this group.

## CONCLUSIONS

In conclusion, an 8-week VBT program carried out in the BP exercise for intermediate resistance-trained women induced different adaptations on the load-velocity relationship depending on the magnitude of VL employed. Specifically, higher VL thresholds (i.e., VL50) led to greater strength gains with heavy loads and muscle hypertrophy. Moderate VL thresholds (i.e., VL25) resulted in positive neuromuscular adaptations during high-velocity lifting and improved the ability to generate force quickly. Additionally, performing only one repetition per set (VL0) at 70–85% 1RM twice a week was sufficient to improve strength in intermediate-trained women but insufficient for maximizing strength gains, particularly under heavy loads. Therefore, unlike in young men, resistance training in intermediatetrained women may require higher training volume and fatigue to maximize strength gains in BP. Nevertheless, high volume and fatigue training may compromise positive neuromuscular adaptations and high-velocity performance.

Several study limitations should be considered when interpreting our results. The present study analyzed only the changes in surface EMG of the TB. However, other prime mover muscles, such as the pectoralis major, were not assessed due to ethical and methodological considerations related to recording EMG activity from the pectoralis major muscle in women, particularly given the anatomical and physiological characteristics unique to the female chest. Additionally, although all participants had at least one year of resistance training experience, they were not athletes.

## Practical applications

For coaches and trainers working with intermediate-trained women, tailoring resistance training programs with VBT principles can optimize different performance outcomes. Higher VL thresholds, such as VL50, may be more effective when the goal is to maximize strength and muscle hypertrophy. However, moderate VL thresholds (i.e., VL25) are preferable for athletes seeking to improve rapid force production and strength deficit, as they promote adaptations that support higher velocity lifting. Additionally, incorporating extremely low-volume strategies, such as performing one repetition per set (VL0) twice a week, can still improve strength, although more is needed for maximizing gains with heavy loads. Practitioners should carefully balance training volume and intensity to avoid compromising neuromuscular adaptations and high-velocity performance.
